# Projection Word Embedding Model With Hybrid Sampling Training for Classifying ICD-10-CM Codes: Longitudinal Observational Study

**DOI:** 10.2196/14499

**Published:** 2019-07-23

**Authors:** Chin Lin, Yu-Sheng Lou, Dung-Jang Tsai, Chia-Cheng Lee, Chia-Jung Hsu, Ding-Chung Wu, Mei-Chuen Wang, Wen-Hui Fang

**Affiliations:** 1 Graduate Institute of Life Sciences National Defense Medical Center Taipei Taiwan; 2 School of Public Health National Defense Medical Center Taipei Taiwan; 3 Planning and Management Office Tri-Service General Hospital National Defense Medical Center Taipei Taiwan; 4 Department of Medical Record Tri-Service General Hospital National Defense Medical Center Taipei Taiwan; 5 Department of Family and Community Medicine Tri-Service General Hospital National Defense Medical Center Taipei Taiwan

**Keywords:** word embedding, convolutional neural network, artificial intelligence, natural language processing, electronic health records

## Abstract

**Background:**

Most current state-of-the-art models for searching the International Classification of Diseases, Tenth Revision Clinical Modification (ICD-10-CM) codes use word embedding technology to capture useful semantic properties. However, they are limited by the quality of initial word embeddings. Word embedding trained by electronic health records (EHRs) is considered the best, but the vocabulary diversity is limited by previous medical records. Thus, we require a word embedding model that maintains the vocabulary diversity of open internet databases and the medical terminology understanding of EHRs. Moreover, we need to consider the particularity of the disease classification, wherein discharge notes present only positive disease descriptions.

**Objective:**

We aimed to propose a projection word2vec model and a hybrid sampling method. In addition, we aimed to conduct a series of experiments to validate the effectiveness of these methods.

**Methods:**

We compared the projection word2vec model and traditional word2vec model using two corpora sources: English Wikipedia and PubMed journal abstracts. We used seven published datasets to measure the medical semantic understanding of the word2vec models and used these embeddings to identify the three–character-level ICD-10-CM diagnostic codes in a set of discharge notes. On the basis of embedding technology improvement, we also tried to apply the hybrid sampling method to improve accuracy. The 94,483 labeled discharge notes from the Tri-Service General Hospital of Taipei, Taiwan, from June 1, 2015, to June 30, 2017, were used. To evaluate the model performance, 24,762 discharge notes from July 1, 2017, to December 31, 2017, from the same hospital were used. Moreover, 74,324 additional discharge notes collected from seven other hospitals were tested. The F-measure, which is the major global measure of effectiveness, was adopted.

**Results:**

In medical semantic understanding, the original EHR embeddings and PubMed embeddings exhibited superior performance to the original Wikipedia embeddings. After projection training technology was applied, the projection Wikipedia embeddings exhibited an obvious improvement but did not reach the level of original EHR embeddings or PubMed embeddings. In the subsequent ICD-10-CM coding experiment, the model that used both projection PubMed and Wikipedia embeddings had the highest testing mean F-measure (0.7362 and 0.6693 in Tri-Service General Hospital and the seven other hospitals, respectively). Moreover, the hybrid sampling method was found to improve the model performance (F-measure=0.7371/0.6698).

**Conclusions:**

The word embeddings trained using EHR and PubMed could understand medical semantics better, and the proposed projection word2vec model improved the ability of medical semantics extraction in Wikipedia embeddings. Although the improvement from the projection word2vec model in the real ICD-10-CM coding task was not substantial, the models could effectively handle emerging diseases. The proposed hybrid sampling method enables the model to behave like a human expert.

## Introduction

Most medical information is recorded as unstructured data [1]. For example, approximately 96% of cancer diagnoses are reported in pathology reports, but are recorded as free-text narrative or images [2]. Disease coding is a common practical data structuralization method that is critical in many fields such as disease surveillance [3], health services management [4], and clinical research [5]. The coding quality can still be improved, and computer-aided coding systems have been considered to increase the accuracy [6,7]. Numerous models have been implemented in recent years [8-11], but they were considered inapplicable [2]. These methods are based on traditional natural language processing (NLP), and their performance is limited by an incomplete medical dictionary. However, compiling a complete medical dictionary may be impossible because of the variability of clinical vocabularies; this is a major challenge for the effective use of electronic health records (EHRs) [12].

With the third artificial intelligence revolution started by the AlexNet win in 2012 [13], further complex deep-learning models such as VGGNet [14], Inception Net [15], ResNet [16], and DenseNet [17] have been developed to achieve performance improvement. The deep-learning model can automatically extract a large amount of useful features to use for prediction [16,18,19]. More than 300 contributions have successfully applied deep-learning technology in medical image analysis [20]. Apart from image analysis, excellent results have been achieved in NLP tasks such as semantic parsing [21], search query retrieval [22], and sentence classification [23]. This has prompted us to develop an artificial intelligence–based model to assist in disease coding in order to achieve faster and more accurate coding.

Word embedding has been prevalently used in current NLP applications. An effective word embedding model is a major breakthrough feature-learning technique where vocabularies are mapped to vectors of real numbers [24-26]. The most popular word embedding models, such as word2vec [26], currently need large free-text resources. Most studies have used two main resources to train the word embedding model for biomedical NLP applications: internal task corpora (eg, EHR) and external internet data resources (eg, Wikipedia). Two studies have evaluated the training of word embedding models using different textual resources for biomedical NLP applications and revealed that the word embedding trained using EHR may capture semantic properties better than that trained using Wikipedia [27,28]. However, Wikipedia has an advantage, which is often overlooked: Its vocabulary diversity of external internet data resources is significantly greater than that of internal task corpora. This advantage has a major effect in real-world disease coding tasks. For example, severe acute respiratory syndrome (SARS) only broke out in 2003 and could not have been recorded in other years. Hence, the word embedding model trained using only internal corpora could not capture the semantic properties of SARS, whereas the internet resources have preserved SARS-related records. The disease coding model applied in the real world should be able to handle emerging diseases; for this purpose, most disease coding tasks are still carried out by human experts who can learn from external resources. Thus, there is a need to develop a word embedding training process that maintains the vocabulary diversity of internet resources and incorporates the medical terminology understanding of internal task corpora.

In addition to the influence of word embedding, the subsequent machine learning model also plays a key role in classification accuracy. Word embedding combined with a convolutional neural network (CNN) exhibited outstanding performance compared with traditional methods [29]. However, its performance is still deficient compared with human experts. Studies have designed rule-based approaches for conducting disease coding, which have demonstrated superior performance [8,30]. Upon carefully observing the keyword list presented in these papers, we found that the number of positive terms is more than the number of negative terms. This is an important characteristic to be considered in the design of a model for imitating human experts. However, rule-based approaches in the development of the disease coding model are expensive. To the best of our knowledge, no methods have been proposed to prevent the machine-learning model from identifying negative terms.

We propose a projection word2vec model to solve the limitation of vocabulary size in EHRs by incorporating internet sources and a hybrid sampling training method that avoids negative term identification. An experiment involving 193,647 discharge notes was conducted to verify the effectiveness. The primary aim of this experiment was to identify three–character-level International Classification of Diseases, Tenth Revision, Clinical Modification (ICD-10-CM) diagnostic codes in the discharge notes.

## Methods

### Word Embedding

Word embedding technology is useful for integrating synonyms; word2vec [26] is the most popular word embedding model. In this study, we used two internet corpora—English Wikipedia and PubMed journal abstracts—and an internal task corpus—the EHRs of discharge notes. Wikipedia is an encyclopedia that is a written compendium of knowledge. PubMed is a free biomedical and life science resource developed and maintained by the National Center for Biotechnology Information, and more than 27 million journal articles have been published as of January 1, 2017. The EHRs used in this study were obtained from Tri-Service General Hospital, Taipei, Taiwan, and the details of these databases are described in the subsequent section. The three corpora were used to train the traditional word2vec model.

A recent word embedding comparison study demonstrated that word embedding trained using EHRs can usually better capture medical semantics [27]. However, the total number of words in our EHRs was only approximately 30,000, which is considerably less than those in the English Wikipedia (~365,000) and PubMed journal abstracts (~375,000). This difference was also present in previous studies, despite a larger data volume in their EHRs [27,28]. This is due to the absence of some rare diseases and periodic diseases in the database, for example, SARS outbreak in 2003 and H1N1 influenza outbreak in 2009. Thus, the word embedding model trained using EHRs cannot include sufficient vocabularies, and the subsequent machine learning model cannot handle diseases not present in the internal database. Thus, we sought to develop a word embedding training process that can maintain the vocabulary diversity of Wikipedia/PubMed and the medical semantic understanding of EHRs.

**Figure 1:**
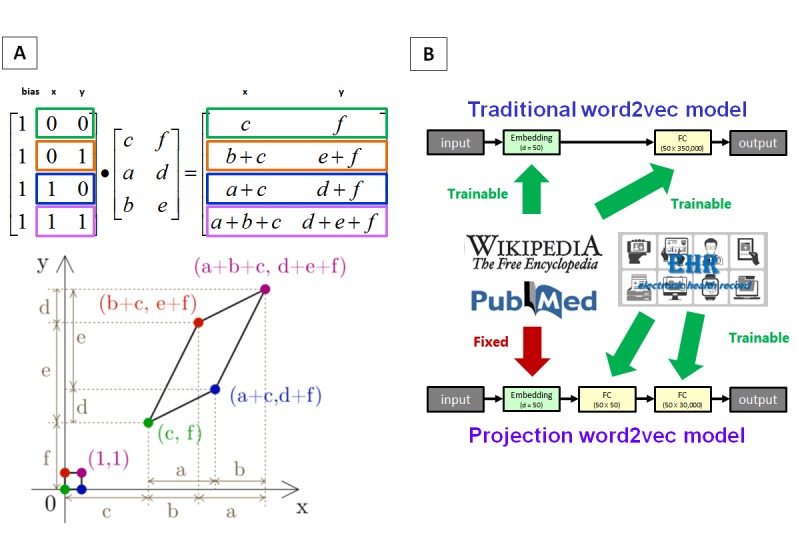
Concept of the projection word embedding model.

The basic concept is presented in Figure 1A. The linear algebra projection is based on matrix multiplication, and all coordinates can be transformed into a new coordinate system. This conversion changes the relevance of some points but maintains all existing coordinates simultaneously. The example presented in Figure 1A indicates that the distance between the original green point and blue point is equal to the distance between the original green point and orange point, but their relationships have changed after projection. Using this method, we revised the traditional word2vec model, as presented in Figure 1B. The traditional word2vec model has two trainable layers, and the embedding weights can be used to express the terminology meanings. Here, we added a convolutional operator after the embedding layer to realize the projection word2vec model. The training process of this projection word2vec model was as follows: (1) the traditional word2vec model was trained by larger internet corpora (ie, Wikipedia and PubMed) and (2) the embedding layer was fixed and a projection word2vec model was trained by the smaller internal corpus (ie, EHRs). The detailed projection word2vec model architecture started from an embedding layer, followed by a fully connected layer for linear projection. Subsequently, another fully connected layer was followed by the linear projection output. The output layer was a logistic output with a noise contrastive estimation loss function.

We used the MXNet version 1.3.0 open-source package to implement these word2vec models. The training parameters of traditional and projection word2vec models employed default settings [26] as follows: skip-gram architecture, a window size of 12, a dimension of 50, a minimum word frequency of 20, a negative sampling parameter of 5, a learning rate of 0.1, and a momentum of 0.9. The well-trained projection Wikipedia/PubMed embeddings can be downloaded from Multimedia Appendix 1.

Because the projection Wikipedia/PubMed embeddings were actually trained by one of the open internet databases and EHRs, we additionally used two combinations of embeddings—original EHR+Wikipedia embeddings and original EHR+PubMed embeddings—as the baseline comparison. The method of combination is a simple concatenation of two vectors, so the length of the vector will be changed to 100. However, the simple concatenation cannot increase the vocabulary size; therefore, we will only compare the performance of the simple combination and our projection word2vec model in medical semantic understanding.

### Medical Semantic Understanding Evaluation

We used the following seven published datasets to measure semantic similarity between medical terms: Hliaoutakis [31], MayoSRS [32], MiniMayoSRS [33,34], UMNSRS-Relatedness [35], UMNSRS-Relatedness-MOD [28], UMNSRS-Similarity [35], and UMNSRS-Similarity-MOD [28]. These databases provided the relevance of each medical term assessed by experts. For example, a relation score of 391 for the terms “cataracts” and “insulin” and a score of 1142 for the terms “obesity” and “diabetes” indicated that the similarity of the second pair was higher. We used different word embedding models for these term pairs and compared the correlation of the word embedding model and original data. The relation scores of each word embedding model were defined as the cosine similarity. If the number of words in a term was more than one, the average vector value from a previous study was used [27]. When the word that needed to be compared did not have any embedding, we chose the most similar word based on a character-level comparison to replace it in order to obtain its embeddings.

In addition to qualitative data, we also selected the following five words, which are the most common diseases in our EHRs, to determine corresponding similar words in different word embeddings: neoplasm, hypertension, diabetes, pneumonia, and sepsis. The cosine similarity was again used to calculate the semantic similarity of these words. The top five most similar words were shown to provide qualitative evidence for measuring the performance of each word2vec model.

### Discharge Note Database

The Tri-Service General Hospital supplied de-identified free-text discharge notes from June 1, 2015, to December 31, 2017. Research ethics approval was issued by the Institutional Ethical Committee and medical records office of the Tri-Service General Hospital to collect data without individual consent for sites where data are directly collected (institutional review board no. 1-107-05-097). The details of this hospital have been described previously [29]. We collected 119,315 discharge notes from the hospital and corrected misspellings using the R hunspell version 2.3 package developed by Jeroen Ooms. Discharge notes are often labeled with multiple ICD-10-CM codes, and in this study, all ICD-10-CM codes were truncated at the three-character level. Table 1 presents the frequency distribution of one–character-level codes. Because of the policy change that entailed the 20th level-1 category, V00-Y99, which was not needed after 2017, we excluded the three–character-level codes in the 20th level-1 category. We divided the sample by date and ensured their proportion to be 0.7, 0.1, and 0.2 in the training, validation, and testing sets, respectively. A classifier can only be trained using retrospective data in the real world, and it is then used to classify future data. Moreover, this study included data from seven hospitals (namely, Taichung Armed Forces General Hospital, Taoyuan Armed Forces General Hospital, Taichung Armed Forces General Hospital Zhongqing Branch, Hualien Armed Forces General Hospital, Tri-Service General Hospital Penghu Branch, Tri-Service General Hospital SongShan Branch, and Zuoying Branch of Kaohsiung Armed Forces General Hospital). The second testing set used 74,324 labeled discharge notes collected from these seven hospitals.

**Table 1:** Prevalence of different one–character-level International Classification of Diseases, Tenth Revision, Clinical Modification codes used in discharge notes in this study.

ICD-10-CM^a^ code	Definition	Dataset			
		Training set^b^ (n=82,390), n (%)	Validation set^c^ (n=12,145), n (%)	Testing set 1^d^ (n=24,780), n (%)	Testing set 2^e^ (n=74,332), n (%)
A00-B99	Certain infectious and parasitic diseases	14,883 (18.1)	2296 (18.9)	4713 (19)	14,704 (19.8)
C00-D49	Neoplasms	29,125 (35.4)	4405 (36.3)	8721 (35.2)	7220 (9.7)
D50-D89	Diseases of the blood and blood-forming organs and certain disorders involving the immune mechanism	8707 (10.6)	1062 (8.7)	2258 (9.1)	7112 (9.6)
E00-E89	Endocrine, nutritional, and metabolic diseases	22,884 (27.8)	3404 (28)	6915 (27.9)	21,866 (29.4)
F01-F99	Mental, behavioral, and neurodevelopmental disorders	7410 (9)	1084 (8.9)	2237 (9)	9956 (13.4)
G00-G99	Diseases of the nervous system	7200 (8.7)	987 (8.1)	2270 (9.2)	5332 (7.2)
H00-H59	Diseases of the eye and adnexa	3039 (3.7)	430 (3.5)	865 (3.5)	873 (1.2)
H60-H95	Diseases of the ear and mastoid process	1044 (1.3)	174 (1.4)	312 (1.3)	846 (1.1)
I00-I99	Diseases of the circulatory system	29,152 (35.4)	4129 (34)	8857 (35.7)	28,509 (38.4)
J00-J99	Diseases of the respiratory system	15,455 (18.8)	2068 (17)	4602 (18.6)	22,344 (30.1)
K00-K95	Diseases of the digestive system	20,621 (25)	2969 (24.4)	5956 24)	22,500 (30.3)
L00-L99	Diseases of the skin and subcutaneous tissue	4217 (5.1)	702 (5.8)	1347 (5.4)	5297 (7.1)
M00-M99	Diseases of the musculoskeletal system and connective tissue	12,030 (14.6)	1697 (14)	3525 (14.2)	10,801 (14.5)
N00-N99	Diseases of the genitourinary system	19,454 (23.6)	2782 (22.9)	5934 (23.9)	18,345 (24.7)
O00-O9A	Pregnancy, childbirth, and the puerperium	2195 (2.7)	311 (2.6)	632 (2.6)	1409 (1.9)
P00-P96	Certain conditions originating in the perinatal period	840 (1)	106 (0.9)	179 (0.7)	375 (0.5)
Q00-Q99	Congenital malformations, deformations, and chromosomal abnormalities	1104 (1.3)	152 (1.3)	286 (1.2)	444 (0.6)
R00-R99	Symptoms, signs, and abnormal clinical and laboratory findings, not elsewhere classified	11,029 (13.4)	1636 (13.5)	3335 (13.5)	13,027 (17.5)
S00-T88	Injury, poisoning, and certain other consequences of external causes	9949 (12.1)	1539 (12.7)	3239 (13.1)	14,244 (19.2)
V00-Y99	External causes of morbidity	114 (0.1)	4 (<0.1)	4 (<0.1)	12,548 (16.9)
Z00-Z99	Factors influencing health status and contact with health services	24,819 (30.1)	4107 (33.8)	8353 (33.7)	15,346 (20.6)

^a^ICD-10-CM: International Classification of Diseases, Tenth Revision, Clinical Modification.

^b^Training set includes samples collected between June 1, 2015, and March 22, 2017, from the Tri-Service General Hospital.

^c^Validation set 1 includes samples collected between March 23, 2017, and June 30, 2017, from the Tri-Service General Hospital.

^d^Testing set 1 includes samples between July 1, 2017, and December 31, 2017, from the Tri-Service General Hospital.

^e^Testing set 2 includes samples from the Taichung Armed Forces General Hospital, Taoyuan Armed Forces General Hospital, Taichung Armed Forces General Hospital Zhongqing Branch, Hualien Armed Forces General Hospital, Tri-Service General Hospital Penghu Branch, Tri-Service General Hospital SongShan Branch, and Zuoying Branch of Kaohsiung Armed Forces General Hospital.

### Artificial Intelligence Model

**Figure 2:**
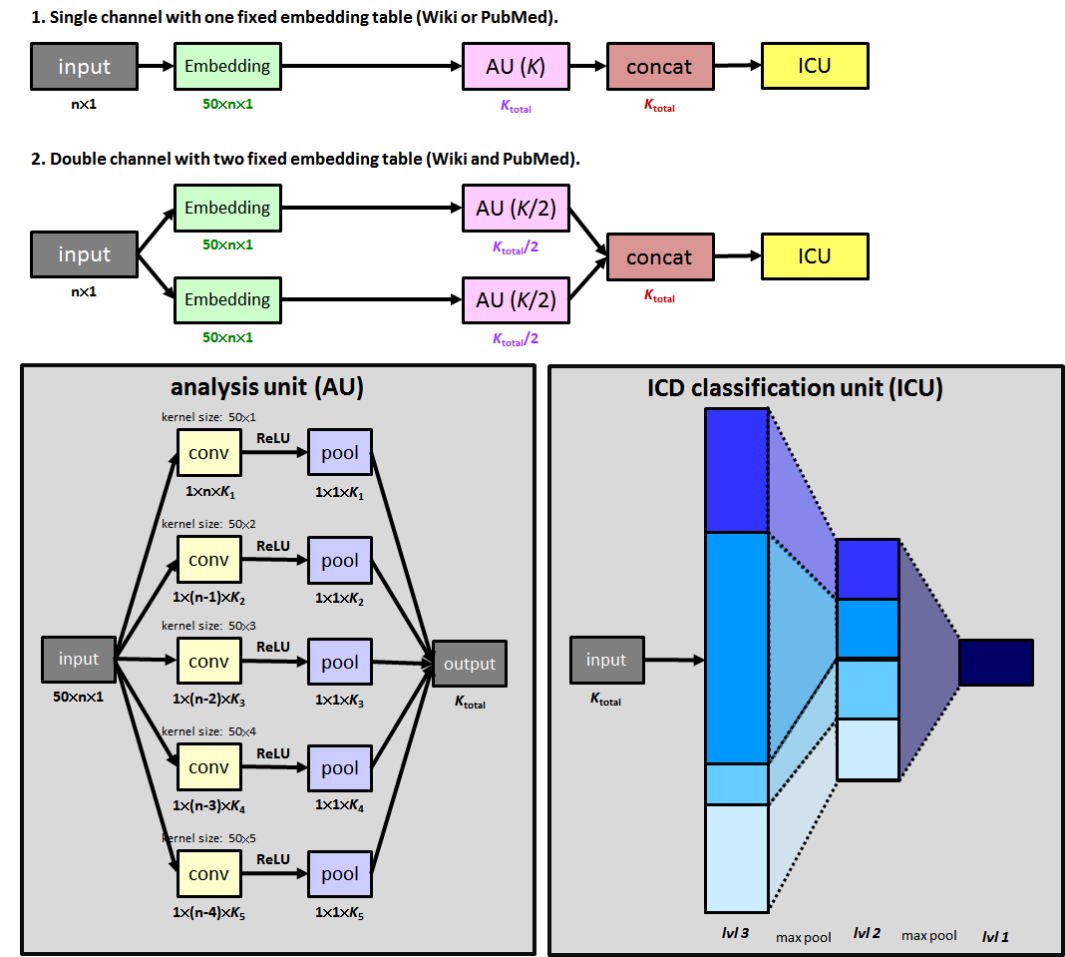
Model architectures in our experiments. ICD: International Classification of Diseases.

One study proposed a model combining a word embedding model and a CNN, which exhibited outstanding performance compared with traditional methods [29]. Here, we used the aforementioned model architecture and revised part of the embedding layer on the basis of our projection word2vec model. Figure 2 shows the details of the model architecture. The input data is an n×1 word sequence, which is converted to a 50×n×1 matrix through a designated embedding table. Subsequently, this matrix is analyzed by our analysis unit, and the output is a vector. The analysis unit is a five-channel coevolution with a filter region size of 1-5 for the disease coding task developed in a previous paper [29]. Here, we slightly revised the architecture for adapting the three­–character-level ICD-10-CM classification task. The convolution channels with 1-5 filter regions have *K*_1_, *K*_2_, *K*_3_, *K*_4_, and *K*_5_ filters, respectively, and *K*_total_ represents the sum of the number of these filters. Figure 2 shows that *K*_total_ is different in each experiment, to ensure that the total number of parameters is the same in all models. For example, in the double-channel model with *K*_total_/2 filters in its analysis unit, the filters are concatenated for the subsequent prediction. In our experiment, we designed *K*_1_, *K*_2_, *K*_3_, *K*_4_, and *K*_5_ to be 2400, 1800, 900, 600, and 300, respectively, in the one-channel model.

Another revision of the previous model is the ICD classification unit. In this study, to extend our model to identify three–character-level ICD-10-CM codes, the number of outputs of the first logistic output layer was revised to the number of the three–character-level ICD-10-CM codes in different one–character-level ICD-10-CM codes. For example, the “Neoplasms” classifier includes 141 outputs, each representing its three–character-level ICD-10-CM code. Subsequently, these output probabilities pass the maximum pooling-layer grouping by their specific two–character-level ICD-10-CM codes, followed by a maximum pooling layer for the one–character-level ICD-10-CM code identification.

Seven different embedding situations can be used to test each performance. Situation a is the baseline setting in which we used EHR embeddings to train the coding model. In situations b and c, embeddings trained from the internet resources Wikipedia and PubMed were used. These models are presented in the first architecture in Figure 2. Situation d is an integrated model that includes the two abovementioned models, as shown in the second architecture in Figure 2. This design was used because of the finding that the vocabularies are highly inconsistent in Wikipedia and PubMed. Because only approximately 100,000 words are included in both Wikipedia and PubMed, this design may help the model recognize more words. Situations e and f are similar to situations b and c, but with the projection Wikipedia and PubMed embeddings used to replace the embedding parameters. Finally, situation g is also an integrated model combining situations e and f.

We used the R MXNet version 1.3.0 package developed by Distributed (Deep) Machine Learning Community to implement the aforementioned architecture. The settings used for the training model are based on our previous paper [29] as follows: the stochastic gradient descent optimizer with 0.05 initial learning rate and 32 bench size for optimization, a weight decay of 10^−4^ [36], a Nesterov momentum [37] of 0.9 without dampening, and the learning rate lowered by 10 three times when validation loss plateaus after an epoch. The cross-entropy was used as the loss function in this study. Because oversampling was adopted for rare categories to improve the model performance [38], we weighed the benefits of cross-entropy on the basis of the frequency of each code. The F-measure was the major evaluation index in our study and is calculated as follows:

*Precision=true positives/true positives+false positives*

*Recall=true positives/true positives+false negatives*

*F-measure=(2×precision×recall)/(precision+recall).*

Moreover, the precision and recall values are provided.

### Hybrid Sampling Training Method

**Figure 3:**
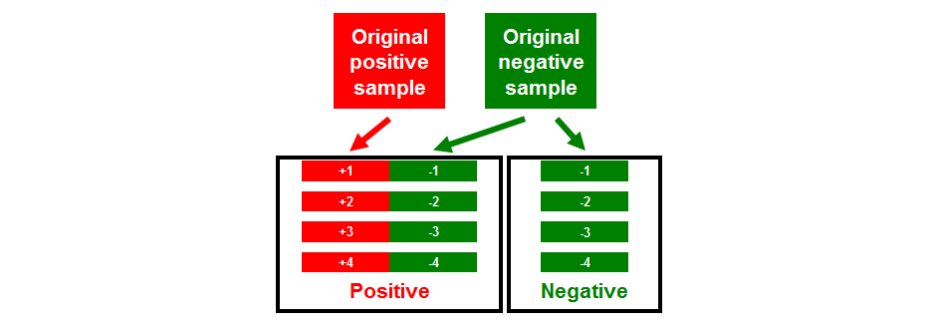
Hybrid sampling method.

A novel ICD-10-CM-specific augmentation method called “hybrid sampling” is proposed for improving model performance. Figure 3 shows the practical details. Data augmentation is a key method for avoiding overfitting and is widely used in the ImageNet Large Scale Visual Recognition Challenge (ILSVRC) [13]. With regard to the disease coding task of discharge notes, the negative terms are useless because the discharge notes include only positive disease descriptions. Thus, a successful training process needs to prevent the model from learning negative terms. The hybrid sampling is based on the hybridization of positive and negative samples. We paste the positive discharge note and a random negative discharge note as a new positive sample for model training, which will disrupt the correlation between keywords. For example, pregnancy-related terms rarely appear in cancer-related discharge notes; hence, the machine-learning model training by the traditional process will discover that the pregnancy-related terms are negative terms for the cancer identification task. However, this is logically incorrect. If human experts consider a discharge note not involving cancer, they will verify that there are no cancer-related terms after carefully reading all descriptions. Hybrid sampling may solve this problem by letting our model only identify positive terms.

## Results

We tested word embeddings on seven published biomedical measurement datasets commonly used to measure the semantic similarity between medical terms. Table 2 lists the Pearson correlation coefficient results for the seven datasets. For Hliaoutakis’ dataset [31], consisting of 34 medical term pairs with similarity scores obtained by human judgments, the previous study resulted in correlation coefficients of 0.482, 0.311, and 0.247 in EHRs, PubMed, and Wikipedia, respectively [27]. Our results are similar, with correlation coefficients of 0.4815, 0.4968, and 0.2820 in original EHRs, PubMed, and Wikipedia embeddings, respectively. The correlation coefficients of the combination of EHR and Wikipedia are between coefficients of the two of them (0.3488), and the combination of EHR and PubMed also shows a similar trend (0.4914). After the projection word2vec training, the correlation coefficients of PubMed and Wikipedia embeddings increased to 0.5255 and 0.3202, respectively. The performances of the simple concatenation and projection model are similar, but the projection model can maintain vocabulary diversity while simple concatenation cannot. The MayoSRS dataset [32] consists of 101 clinical term pairs whose relatedness was determined by nine medical coders and three physicians from the Mayo Clinic, whereas MiniMayoSRS, which is a subset of MayoSRS, includes 29 of 101 term pairs. The previous study demonstrated that the highest correlations of 0.412 and 0.632, respectively, were found in EHR embeddings [27]. Our EHR embeddings also yielded the highest correlation of 0.6082 in MayoSRS, and after the projection word2vec model, the correlations of PubMed and Wikipedia embeddings increased from 0.5087 to 0.5148 and from 0.0082 to 0.0930, respectively.

**Table 2:** Pearson correlation coefficients between similarity scores of disease coding performed by human judgment and those calculated using four-word embeddings.

Series and dataset			Embeddings												
			Original Wikipedia		Original PubMed		Original EHR^a^		Original EHR+Wikipedia		Original EHR+PubMed		Projection Wikipedia		Projection PubMed
**MeSH^b^**															
	Hliaoutakis’	0.2820		0.4968		0.4815		0.3488		0.4914		0.3202		0.5255	
**MayoSRS^c^series**															
	MayoSRS	0.0082		0.5087		0.6082		0.1948		0.6028		0.0930		0.5148	
	MiniMayoSRS	0.3363		0.7200		0.6613		0.4746		0.7201		0.4709		0.5903	
**UMNSRS^d^series**															
	UMNSRS Relatedness	0.2836		0.4891		0.4525		0.3808		0.4774		0.3378		0.4390	
	UMNSRS Relatedness - MOD^e^	0.2985		0.5094		0.5020		0.4015		0.5184		0.3678		0.4903	
	UMNSRS Similarity	0.3032		0.4916		0.4617		0.3906		0.4868		0.3281		0.4071	
	UMNSRS Similarity - MOD	0.3379		0.5271		0.4993		0.4304		0.5272		0.3733		0.4771	

^a^EHR: electronic health record.

^b^MeSH: Medical Subject Headings.

^c^MayoSRS: Mayo Medical Coders Set.

^d^UMNSRS: University of Minnesota Semantic Relatedness Set.

^e^MOD: modification.

However, the original PubMed embeddings yielded the highest correlation of 0.7200 in MiniMayoSRS; hence, the projection word2vec model successfully improved the performance of only Wikipedia embeddings (PubMed: 0.7200→0.5903; Wikipedia: 0.3363→0.4709). The simple concatenation embeddings look slightly better than projection embeddings in these two datasets but are still limited by the vocabulary size of EHRs. This situation was the same for the following four similar datasets: UMNSRS-Relatedness [35], UMNSRS-Relatedness-MOD [28], UMNSRS-Similarity [35], and UMNSRS-Similarity-MOD [28]. The projection word2vec model improved the performance of Wikipedia embeddings but not that of PubMed embeddings because the performance of original PubMed embeddings was higher than that of the original EHR embeddings. The simple concatenation embeddings are still slightly better than projection embeddings. In summary, the proposed projection word2vec model has the potential to improve the performance of capturing semantic properties when the embeddings trained from the original corpus are worse than those from the target corpus. The details of all term pair comparisons are provided in Multimedia Appendix 1.

In the qualitative evaluation, we selected five medical words because they are most common disorders in our discharge notes: neoplasm, hypertension, diabetes, pneumonia, and sepsis. Word embeddings trained from one internal corpus and two internet corpora were utilized to compute the five most similar words to each selected medical word according to the cosine similarity; the results are listed in Table 3. Similar to the quantitative results, an obvious superiority of PubMed/EHR embeddings compared with Wikipedia embeddings was observed when using the traditional word2vec model. For example, the word most similar to “hypertension,” given by PubMed embeddings, was “hypertensive,” which is the adjective of the original word; this was also present in the result of EHR embeddings. In contrast, the first five words most similar to “hypertension” as per the Wikipedia embeddings were all less relevant. However, the performance of the projection Wikipedia embedding model exhibited no obvious improvement compared with the original Wikipedia embedding model. The only notable improvement in the case of the word “hypertension” was the removal of the word “asthma” in the most similar list, which is an obvious unrelated term. This phenomenon was also present in other selected words. Moreover, the results of simple concatenation embeddings resemble those of combining the first five words of two embeddings and reordering them. Because the performance of the original PubMed and EHR embeddings was similar, there was no apparent improvement in the projection technology results compared with the original PubMed embeddings. In summary, we considered the qualitative and quantitative analyses results to be similar.

**Table 3:** Selected words and the corresponding five most similar words obtained from different word embedding models.

Target word	Embeddings						
	Original Wikipedia	Original PubMed	Original EHR^a^	Original EHR+Wikipedia	Original EHR+PubMed	Projection Wikipedia	Projection PubMed
Neoplasm	Malignant	Leiomyosarcoma	Neoplasms	Neoplasms	Neoplasms	Polyp	Angiosarcoma
	Polyp	Angiosarcoma	Carcinoid	Mucinous	Carcinoid	Mucinous	Leiomyosarcoma
	Neoplasms	Malignancy	Lymphoepithelial	Malignant	Mucinous	Malignant	Lipoma
	Nematode	Malignant	Oncocytoma	Pheochromocytoma	Paraganglioma	Nematode	Acinic
	Mucinous	Neoplasms	Mucinous	Carcinoid	Oncocytoma	Cyst	Malignancy
Hypertension	Diabetes	Hypertensive	Hyperlipidemia	Diabetes	Hypertensive	Diabetes	Hypertensive
	Pulmonary	Renovascular	Dyslipidemia	Cardiovascular	Hyperlipidemia	Pulmonary	Dyslipidemia
	Cardiovascular	Cardiovascular	Hypertensive	Chronic	Dyslipidemia	Chronic	Mellitus
	Asthma	Normotension	HCVD	Pulmonary	Cardiovascular	Disease	Hyperlipidemia
	Chronic	Dyslipidemia	Hyperuricemia	Asthma	Hypercholesterolemia	Acute	Dyslipidemia
Diabetes	Hypertension	Mellitus	Mellitus	Hypertension	Mellitus	Hypertension	Mellitus
	Cancer	Diabetic	DM	Cardiovascular	Diabetics	Disease	Diabetics
	Asthma	Diabetics	Diabetics	Diabetics	Diabetic	Patients	Diabetic
	Obesity	Dyslipidemia	Diabetes	Mellitus	NIDDM	Hepatitis	IGT
	Alzheimer	Hyperlipidemia	Cardiovascular	Diabetic	Macrovascular	Treating	Nondiabetic
Pneumonia	Respiratory	Pneumonias	Acquired	Respiratory	Pneumonias	Illness	Pneumonias
	Illness	Bronchopneumonia	Community	Infection	Bacteremic	Respiratory	Bronchopneumonia
	Complications	Bacteremia	Healthcare	Hospitalized	Bacteremia	Infection	Bacteremia
	Bronchitis	Bacteremic	Aspiration	Infections	Acquired	SARS	Nosocomial
	Infection	Meningitis	Pneumonia	Illness	Bronchopneumonia	Hepatitis	Meningitis
Sepsis	Meningitis	Septic	Septic	Septicemia	Septic	Hepatitis	Septic
	Septicemia	Septicemia	Septicemia	Bacteremia	Bacteremia	Respiratory	Septicemia
	Jaundice	Peritonitis	Coli	Infection	Septicemia	Infection	Bacteremia
	Hepatitis	Polymicrobial	Bacteremia	Septicemia	Polymicrobial	Illness	Meningitis
	Diabetes	Mods	Epiglottitis	Meningitis	Septicemia	Jaundice	Polymicrobial

^a^EHR: electronic health record.

Furthermore, we applied the abovementioned embedding models on the three–character-level ICD-10-CM coding task; Table 4 shows the global means of F-measures of the tests. In the task, the first testing samples were divided according to the date, and the second samples were from the seven other hospitals. Because some three–character-level codes were never or less frequently used, we only present the results of the 90% most used three–character-level ICD-10-CM codes. The usage rates of all included codes were more than 0.2%; this situation was somewhat reversed. The performance of the model trained by PubMed embeddings was worse than that of Wikipedia and EHR embeddings. The model trained by EHR embeddings (0.7250/0.6574) yielded a higher mean of F-measures than Wikipedia embeddings (0.7213/0.6479), followed by the PubMed embeddings (0.6974/0.6260), both in the first and second test sets. It is worth mentioning that the integrated model that used both Wikipedia and PubMed embeddings (0.7208) achieved similar performance to the model that used only Wikipedia embeddings in the first test set but the former showed better performance (0.6540) in the second test set. Therefore, the projection technique showed an improvement on the model performance in all embeddings consistently in all situations (Wiki: 0.7213/0.6479 to 0.7316/0.6617; PubMed: 0.6974/0.6260 to 0.7187/0.6561; Wiki+PubMed: 0.7208/0.6540 to 0.7362/0.6693). The model that used both projection Wikipedia and PubMed embeddings exhibited the best performance compared with all models. However, the model that used projection Wikipedia embeddings was only slightly behind it. The best model, determined on the basis of the comparison of embeddings and, namely, the hybrid sampling method, was used for improving the model performance. Although the improvement was not large, the hybrid sampling training further improved the model performance (0.7371/0.6698). The details of all precisions, recalls, and F-measures are presented in Multimedia Appendix 2.

**Figure 4:**
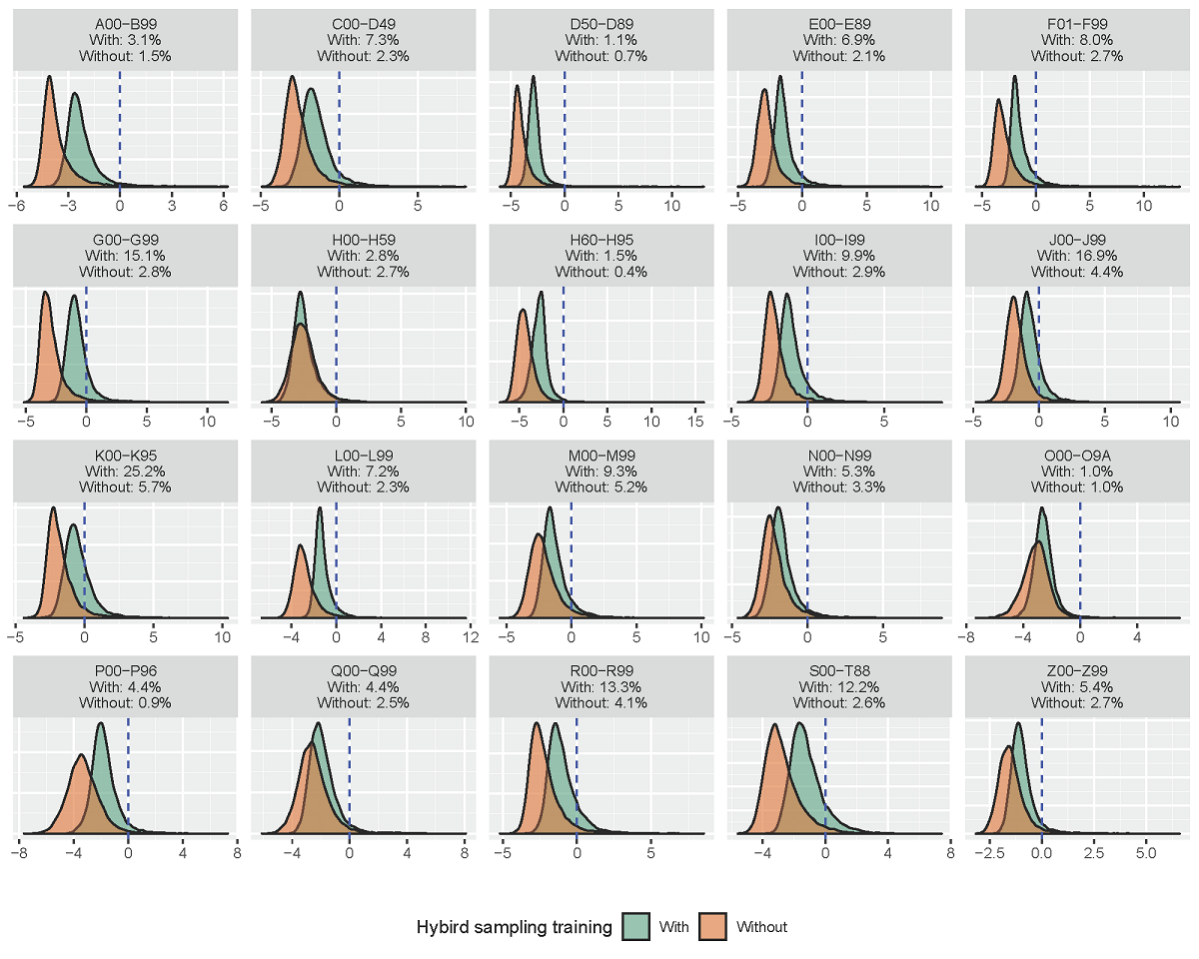
Density plots of predictions of each single word provided by the model with and without hybrid sampling training.

To further understand the effect of hybrid sampling training, we compared the predictions of each word in the model with (situation h in Table 4) and without (situation g in Table 4) hybrid sampling training. We included all words in our EHRs, and Figure 4 presents the density plot of predictive results in 20 one–character-level codes. The prediction values are defined as the last fully connected output before logistic transformation; therefore, a value greater than 0 implies that the model results in a probability greater than 50% for only single–character-level words. The percentage presented in Figure 4 represents the proportion of words with a value more than 0; therefore, a higher value implies that the model often uses positive terms for predictions. It is noteworthy that the model with hybrid sampling training exhibited the highest proportion of positive terms used in all one–character-level codes. We further present the ICD-10-CM identification results of two simulated discharge notes generated by the models with and without hybrid sampling training to further understand the hybrid model’s effect; the results are listed in Table 5. In our discharge notes, we identified a strong negative correlation between cancer and pregnancy; hence, in this experiment, we tried to simulate the discharge notes with cancer and pregnancy. The first case was a primipara with duodenal adenocarcinoma. The model without hybrid sampling training ignored two three–character-level codes: O60 and C17; omission of C17 is unacceptable because it is the main code in this case. The model with hybrid sampling training successfully recognized these codes but also identified an error code, K91. This example clearly indicates that the second model performed better, but the average accuracies of the two models were similar. The second case was another description style by strip format; the model with hybrid sampling training successfully recognized the code C53 again, whereas the model without hybrid sampling training could not. We understand the defects of average F-measures through these two examples. Thus, the hybrid sampling training, in fact, improved the model, although there was only a slight improvement in the average F-measures.

**Table 4:** Results of the three–character-level ICD-10-CM coding task using different word embeddings (italicized font indicates the best precision, recall, and F-measure).

Situations	Testing set 1^a^			Testing set 2^b^		
	Precision	Recall	F-measure	Precision	Recall	F-measure
a: EHR^c^	0.7156	0.7724	0.7250	0.6852	0.6932	0.6574
b: Wikipedia	0.7106	0.7689	0.7213	0.6879	0.6743	0.6479
c: PubMed	0.6723	0.7725	0.6974	0.6491	0.6776	0.6260
d: EHR+Wikipedia	0.7066	0.7665	0.7208	0.6854	0.6797	0.6540
e: Projection Wikipedia	0.7177	0.7776	0.7316	0.6877	0.6929	0.6617
f: Projection PubMed	0.7070	0.7700	0.7187	0.6817	0.6908	0.6561
g: Projection Wikipedia+Projection PubMed	*0.7205*	0.7809	0.7362	*0.6892*	0.6994	0.6693
h: Projection Wikipedia+Projection PubMed+Hybrid sampling	0.7189	*0.7832*	*0.7371*	0.6826	*0.7081*	*0.6698*

^a^Testing set 1 includes the samples collected between July 1, 2017, and December 31, 2017, from the Tri-Service General Hospital.

^b^Testing set 2 includes the samples from the Taichung Armed Forces General Hospital, Taoyuan Armed Forces General Hospital, Taichung Armed Forces General Hospital Zhongqing Branch, Hualien Armed Forces General Hospital, Tri-Service General Hospital Penghu Branch, Tri-Service General Hospital SongShan Branch, and Zuoying Branch of Kaohsiung Armed Forces General Hospital.

^c^EHR: electronic health record.

**Table 5:** ICD-10-CM coding results of selected models in several simulated discharge notes (italicized font indicates inconsistent predictions among the models with and without hybrid sampling training).^a^

Example discharge note and expected result		Hybrid sampling training	
		Without (%)^b^	With (%)^c^
**Pregnancy 36 2/7 weeks with previous cesarean section, delivered by cesarean section; duodenal adenocarcinoma, second portion with ampullar Vater invasion; acute pancreatitis and hepatitis, suspected biliary obstruction related**			
	*C17*	Z3A (100)	O34 (100)
	O34	Z37 (99)	Z37 (100)
	O34	O34 (98)	Z3A (100)
	O34	K85 (97)	K83 (99)
	K85	K75 (96)	K85 (99)
	K75	K83 (95)	K75 (99)
	Z37	N/A^d^	*K91 (78)*
	Z3A	N/A	*C17 (74)*
	N/A	N/A	*O60 (71)*
**Pregnancy 38 4/7 weeks with previous cesarean section, delivered by cesarean section; moderately differentiated adenocarcinoma of cervix**			
	O34	Z37 (99)	O34 (100)
	Z37	Z3A (99)	Z37 (100)
	Z3A	O34 (99)	Z3A (100)
	*C53*	N/A	*C53 (87)*

^a^List of ICD-10-CM codes used: C17: malignant neoplasm of small intestine; O34: maternal care for abnormality of pelvic organs; O60: preterm labor; K83: other diseases of biliary tract; K85: acute pancreatitis; K75: other inflammatory liver diseases; Z37: outcome of delivery; Z3A: weeks of gestation; K91: intraoperative and postprocedural complications and disorders of digestive system, not elsewhere classified; C53: malignant neoplasm of cervix uteri.

^b^The classification model trained by projection Wikipedia and PubMed embeddings (situation g in Table 4).

^c^The classification model trained by projection Wikipedia and PubMed embeddings and hybrid sampling method (situation h in Table 4).

^d^N/A: not applicable.

## Discussion

The EHR embeddings and PubMed embeddings trained by the traditional word2vec model have a similar ability to capture medical semantic properties, and they are better than the Wikipedia embedding model. After the projection word2vec training, the projection Wikipedia embedding exhibited an obvious improvement compared with the original version. In the three–character-level ICD-10-CM coding task, the projection word2vec model performed better, and the model that used both projection Wikipedia and PubMed embeddings was the best of them. Although the proposed “hybrid sampling” method only slightly improved the model performance, it successfully avoided the interference of negative terms. In summary, the proposed projection word embedding model and hybrid sampling training method provide a new opportunity to improve the performance of medical NLP.

The most significant advantage of the proposed projection word2vec model is that it can maintain vocabulary diversity from external internet resources and provide a more accurate understanding of medical semantics from internal resources. Because of the limitations imposed by relevant regulations, such as the Health Insurance Portability and Accountability Act and General Data Protection Regulation, the EHR resources may not be publicly available. This limits the vocabulary size of models trained by EHRs that are owned by research teams. However, previous studies have found that word embeddings trained using EHRs may capture semantic properties better than those trained using Wikipedia [27,28]. A common alternative has been to replace the Wikipedia resource with the PubMed resource, which demonstrates the advantage of PubMed embeddings in medical semantic understanding [27,28]. However, a machine learning model using PubMed embeddings exhibited the worst performance in multiple tasks compared with that using EHR embeddings, because PubMed is a biomedical and life science journal article resource [27]. In our ICD-10-CM coding task, the model using PubMed embeddings performed even worse than that using Wikipedia embeddings. In short, although EHR embeddings are necessary in medical NLP tasks, vocabulary diversity is inevitably restricted because the vocabulary size is less than 100,000 words, even in a large EHR [27,28]. We overcome this problem through the use of the proposed projection word2vec model, and the experimental results demonstrated the superiority of projection Wikipedia and PubMed embeddings. The proposed projection word2vec model can not only deal with the vocabulary size problem in the medical NLP task but also be used in other fields that require confidentiality of data. Thus, the proposed projection word2vec model simultaneously maintains the advantages of both internal and external corpora but does not focus on improving the model performance.

The basic idea of our projection word2vec model is very similar to transfer learning [39], but it is not a direct application because of the particularity of our task. Most transfer learning was initially trained by a large dataset and kept the same architecture to continuously train on a specific domain. However, the vocabulary lists of open internet databases and EHRs are inevitably different, and the embeddings of some vocabulary not included in EHRs will not be changed when we train them by EHRs. This will destroy the semantic relationship in original open internet databases. Our projection design keeps the original embeddings and changes all weights together, and the embeddings of vocabulary not included in EHRs will also be changed by their similar terms included in EHRs. This idea can also be used in other NLP tasks to add to the vocabulary diversity and terminology understanding of their word embeddings.

An unexpected finding in the medical semantic understanding evaluation was that original PubMed embeddings were better than original EHR embeddings; this was because our EHR was smaller than those in previous studies [27,28]. However, only the MayoSRS dataset showed an opposite result. The reason is the different word compositions in these seven datasets. The MayoSRS included more symptom and sign words than the other datasets. Because EHRs describe the medical records with more symptoms and signs than journal articles, the embeddings trained by EHRs are superior in capturing symptom or sign semantics. Moreover, due to the attenuation, performance of the projection PubMed embeddings was worse than both the original EHR embeddings and original PubMed embeddings in MiniMayoSRS and all of the UMNSRS datasets. In our experiment, there was only one additional projection matrix with 2500 parameters for modifying the medical terminology understanding by EHRs, and this is relatively small compared to the number of parameters in original EHR embeddings. Thus, the projection may only be able to enforce a part of the medical terminology understanding. The EHRs used more nondiagnostic and drug words, so the projection model may not correct the understanding of diagnosis and drug words, which is the major issue in UMNSRS databases and MiniMayoSRS. However, the most significant advantage of the projection model is to maintain the vocabulary diversity. Further, the ICD-10-CM coding task shows that projection embeddings are better than original embeddings. Therefore, we believe that this unexpected attenuation may not negatively affect the advantage of the purposed projection model.

Medical semantics learning using PubMed is expected to be better than that using Wikipedia. In the similarity scores test, the PubMed embeddings exhibited a superior ability to capture medical semantic properties compared with Wikipedia embeddings, which is consistent with previous studies [27,28]. However, further machine learning using PubMed embeddings performed worse in the ICD-10-CM coding task compared with Wikipedia embeddings. From a theoretical view, the frequency with which medical terms appear in journal abstracts is higher than that in general articles; hence, their characteristics can be learned better in the PubMed database. The reason for this experimental result is likely that the medical records are still different from journal resources. The model trained using EHRs exhibited the best performance probably because the key points of the three–character-level task were organ names. Only a few medical studies have explored more than one organ; hence, semantic learning from Wikipedia and PubMed has advantages in different situations. We propose a double-channel model that includes both Wikipedia and PubMed embeddings to solve this problem. This model not only improved the vocabulary size because the vocabularies are highly inconsistent in Wikipedia and PubMed but also achieved the best performance in our ICD-10-CM coding experiments. The projection word2vec model can still improve the performance of the double-channel model. Further investigation can follow this design to perform disease coding tasks.

The discharge notes almost only describe the positive statements, and this is very different from other NLP tasks. Most previous rule-based systems list only the positive terms and demonstrate superior performance [8,30]; therefore, designing a method for the model to avoid negative weighting words was crucial. A naive idea was to limit model parameters to positive numbers in the training process. However, current artificial intelligence technology is based on backpropagation, which utilizes gradient transfer and the chain rule, so all mathematical functions used in artificial intelligence models need to be differentiable. Thus, we could not directly limit model parameters to positive numbers. The hybrid sampling method was a breakthrough concept. We designed a soft limit for model parameters through the modification of input data. In further analysis, the model with hybrid sampling used positive words more often. However, the model performance improved only slightly through implementation of the hybrid sampling method in our experiments; this may be due to the similarity of discharge notes between the training set and test set in our experiments. In the subsequent virtual medical records analysis, we tried to simulate medical records that did not appear in our hospital EHRs by using the model with hybrid sampling training, and superior performance was achieved. Although we could not provide qualitative evidence for this improvement, it must be focused upon in further analysis. A fully automatic model applied in practical use should be able to handle this challenge. We expect this technique to be widely used in subsequent disease coding research, and only positive descriptions will be presented for some free-text document classification tasks.

Although the accuracy of disease coding was improved only slightly by our proposed methods, we achieved the best accuracy reported in the literature. Only a few studies have reported the ability to automatically identify three–character-level ICD-10-CM codes from the free-text medical records because of its difficulty. Koopman et al [40] claimed that their model could effectively determine common types of cancers (mean F-measure=0.7) [40], and our model archive discerned a huge lead in the same 20 cancer types (0.7579 in the testing set from the same source). In fact, these 20 cancers are not the first 20 common cancer types in our sample. The mean F-measure in our first 20 common cancer types was 0.8617. This suggests the advantages of our model as well as the success of the modern artificial intelligence model. Existing deep learning models have been proven to achieve human-level performance and to be effective in medical applications where large annotated datasets are available [16,18-20]. Our study integrated state-of-the-art artificial intelligence into the model to easily perform the disease coding task.

This study has several potential limitations. First, we used only a 50-dimension embedding model to process our data. This related small number may also cause additional attenuation in medical terminology understanding, because the number of parameters in the projection matrix is the square of the small number. However, one study presented data processing for the ICD-10-CM coding task [29], and another proposed that a 60-dimension embedding model is better than a 100-dimension embedding model [27]. We consider that the optimal dimension number of embeddings may need more study. Second, the data volume of our EHRs was smaller than that of previous studies,[27,28] which may have affected the performance of EHR embeddings and projection embeddings based on EHR. However, the correlations of our EHR embeddings in the database consisting of seven medical term pairs were not lower than the correlations in these studies [27,28]. Third, this study used only a set of hyperparameters for all model trainings due to limitations of computing resources; hence, the performance can still be improved. However, the model performance was better than that of previously proposed methods. Moreover, this study collected multicenter data sources to validate the model performance. The similarity trends confirmed the robustness of the set of hyperparameters. Therefore, our experimental setting is convincing from the perspective of model research.

In conclusion, in this paper, we proposed a projection word2vec model to use for expressing the meaning of medical terminology with more accuracy, and we confirmed the effectiveness of the architecture in disease classification using free-text discharge notes from hospitals. Moreover, a novel augmentation method—the hybrid sampling method—was proposed to prevent models from identifying negative terms. With the third generation of artificial intelligence revolution initiated in the ILSVRC 2012, the artificial intelligence model is expected to change the health care system. We believe that the projection word2vec model can be applied in discharge note classification as well as other situations. When there is a small high-quality corpus and a large external corpus, the projection word2vec model can help maintain both vocabulary diversity and medical semantic understanding. Future NLP can become more powerful and robust due to the improved performance of the proposed models.

## Appendix

Multimedia Appendix 1 Projection Wikipedia/PubMed embeddings.

Multimedia Appendix 2 Precision, recall, and F-measure values.

## Abbreviations

CNN: convolutional neural network
EHR: electronic health record
ICD-10-CM: International Classification of Diseases, Tenth Revision, Clinical Modification
ILSVRC: ImageNet Large Scale Visual Recognition Challenge
NLP: natural language processing
SARS: severe acute respiratory syndrome

## References

[1] Murdoch TB, Detsky AS (2013). The inevitable application of big data to health care. JAMA.

[2] Spasić I, Livsey J, Keane JA, Nenadić G (2014). Text mining of cancer-related information: review of current status and future directions. Int J Med Inform.

[3] Lee LM, Thacker SB (2011). Public health surveillance and knowing about health in the context of growing sources of health data. Am J Prev Med.

[4] Uzkuraitis C, Hastings K, Torney B (2010). Casemix Funding Optimisation: Working Together to Make the Most of Every Episode. Health Inf Manag.

[5] Ho C, Guilcher SJT, McKenzie N, Mouneimne M, Williams A, Voth J, Chen Y, Cronin S, Noonan VK, Jaglal SB (2017). Validation of Algorithm to Identify Persons with Non-traumatic Spinal Cord Dysfunction in Canada Using Administrative Health Data. Top Spinal Cord Inj Rehabil.

[6] do Nascimento RL, Castilla EE, Dutra MDG, Orioli IM (2018). ICD-10 impact on ascertainment and accuracy of oral cleft cases as recorded by the Brazilian national live birth information system. Am J Med Genet A.

[7] Peng M, Sundararajan V, Williamson T, Minty EP, Smith TC, Doktorchik CTA, Quan H (2018). Exploration of association rule mining for coding consistency and completeness assessment in inpatient administrative health data. J Biomed Inform.

[8] Koopman B, Karimi S, Nguyen A, McGuire R, Muscatello D, Kemp M, Truran D, Zhang M, Thackway S (2015). Automatic classification of diseases from free-text death certificates for real-time surveillance. BMC Med Inform Decis Mak.

[9] Koopman B, Zuccon G, Wagholikar A, Chu K, O'Dwyer J, Nguyen A, Keijzers G (2015). Automated Reconciliation of Radiology Reports and Discharge Summaries. AMIA Annu Symp Proc.

[10] Khachidze M, Tsintsadze M, Archuadze M (2016). Natural Language Processing Based Instrument for Classification of Free Text Medical Records. Biomed Res Int.

[11] Mujtaba G, Shuib L, Raj RG, Rajandram R, Shaikh K, Al-Garadi MA (2017). Automatic ICD-10 multi-class classification of cause of death from plaintext autopsy reports through expert-driven feature selection. PLoS One.

[12] Rajkomar A, Oren E, Chen K, Dai A, Hajaj N, Hardt M, Liu Pj, Liu X, Marcus J, Sun M, Sundberg P, Yee H, Zhang K, Zhang Y, Flores G, Duggan Ge, Irvine J, Le Q, Litsch K, Mossin A, Tansuwan J, Wang D, Wexler J, Wilson J, Ludwig D, Volchenboum Sl, Chou K, Pearson M, Madabushi S, Shah Nh, Butte Aj, Howell Md, Cui C, Corrado Gs, Dean J (2018). Scalable and accurate deep learning with electronic health records. npj Digital Med.

[13] Krizhevsky A, Sutskever I, Hinton G (2012). ImageNet classification with deep convolutional neural networks. https://papers.nips.cc/paper/4824-imagenet-classification-with-deep-convolutional-neural-networks.pdf.

[14] Simonyan K, Zisserman A (2015). Cornell University.

[15] Szegedy C, Liu W, Jia Y, Sermanet P, Reed S, Anguelov D Cornell University.

[16] He K, Zhang X, Ren S, Sun J (2016). Deep Residual Learning for Image Recognition.

[17] Huang G, Liu Z, Weinberger K, van der Maaten L (2017). Densely Connected Convolutional Networks.

[18] Amodei D, Ananthanarayanan S, Anubhai R, Bai J, Battenberg E, Case C Cornell University.

[19] Xiong W, Droppo J, Huang X, Seide F, Seltzer M, Stolcke A Cornell University.

[20] Litjens G, Kooi T, Bejnordi BE, Setio AAA, Ciompi F, Ghafoorian M, van der Laak JAWM, van Ginneken B, Sánchez Clara I (2017). A survey on deep learning in medical image analysis. Med Image Anal.

[21] Yih W, He X, Meek C (2014). Semantic Parsing for Single-Relation Question Answering. http://acl2014.org/acl2014/P14-2/pdf/P14-2105.pdf.

[22] Shen Y, He X, Gao J, Deng L, Mesnil G, editors (2014). Learning semantic representations using convolutional neural networks for web search.

[23] Kim Y (2014). Convolutional Neural Networks for Sentence Classification.

[24] Bengio Y, Ducharme R, Vincent P, Jauvin C (2003). A neural probabilistic language model. The Journal of Machine Learning Research.

[25] Yih W, Toutanova K, Platt J, Meek C (2011). Learning discriminative projections for text similarity measures.

[26] Mikolov T, Sutskever I, Chen K, Corrado G, Dean J (2013). Distributed representations of words and phrases and their compositionality.

[27] Wang Y, Liu S, Afzal N, Rastegar-Mojarad M, Wang L, Shen F, Kingsbury P, Liu H (2018). A comparison of word embeddings for the biomedical natural language processing. J Biomed Inform.

[28] Pakhomov S, Finley G, McEwan R, Wang Y, Melton G (2016). Corpus domain effects on distributional semantic modeling of medical terms. Bioinformatics.

[29] Lin C, Hsu C, Lou Y, Yeh S, Lee C, Su S, Chen H (2017). Artificial Intelligence Learning Semantics via External Resources for Classifying Diagnosis Codes in Discharge Notes. J Med Internet Res.

[30] Muscatello DJ, Morton PM, Evans I, Gilmour R (2008). Prospective surveillance of excess mortality due to influenza in New South Wales: feasibility and statistical approach. Commun Dis Intell Q Rep.

[31] Hliaoutakis A (2005). Semantic Similarity Measures in MeSH Ontology and their application to Information Retrieval on Medline.

[32] Pakhomov S, Pedersen T, McInnes B, Melton G, Ruggieri A, Chute C (2011). Towards a framework for developing semantic relatedness reference standards. J Biomed Inform.

[33] Pedersen T, Pakhomov S, Patwardhan S, Chute C (2007). Measures of semantic similarity and relatedness in the biomedical domain. J Biomed Inform.

[34] McInnes B, Pedersen T, Pakhomov S (2009). UMLS-Interface and UMLS-Similarity : open source software for measuring paths and semantic similarity. AMIA Annu Symp Proc.

[35] Pakhomov S, McInnes B, Adam T, Liu Y, Pedersen T, Melton GB (2010). Semantic Similarity and Relatedness between Clinical Terms: An Experimental Study. AMIA Annu Symp Proc.

[36] Gross S, Wilber M (2016). Torch.

[37] Sutskever I, Martens J, Dahl G, Hinton G (2013). On the importance of initialization and momentum in deep learning.

[38] Simpson A (2015). Cornell University.

[39] Pan S, Yang Q (2010). A Survey on Transfer Learning. IEEE Trans Knowl Data Eng.

[40] Koopman B, Zuccon G, Nguyen A, Bergheim A, Grayson N (2015). Automatic ICD-10 classification of cancers from free-text death certificates. Int J Med Inform.

